# Supplementary material for: Identification of Freezing-Responsive microRNAs and Their Targets in Chinese Jujube by Small RNA and Degradome Sequencing
Source: Int J Mol Sci. 2026 Sep 4;27(17):7912. doi: 10.3390/ijms27177912 (PMC13566890; doi:10.3390/ijms27177912)
Supplement: Supplementary file 1 [file ijms-27-07912-s001.zip › Supplementary Material S2/novel_miR_121.pdf]

Provisional ID	:	novel_miR_121
Score total	:	0.7
Score for star read(s)	:	-1.3
Score for read counts	:	0
Score for mfe	:	0.4
Score for randfold	:	1.6
Score for cons. seed	:	
Total read count	:	1164
Mature read count	:	1129
Loop read count	:	35
Star read count	:	0

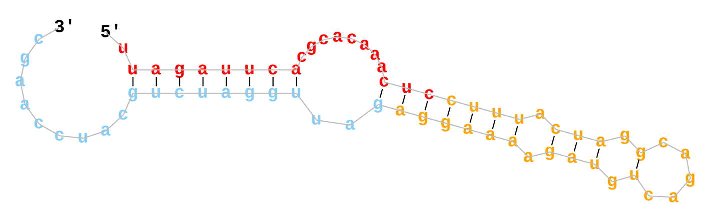

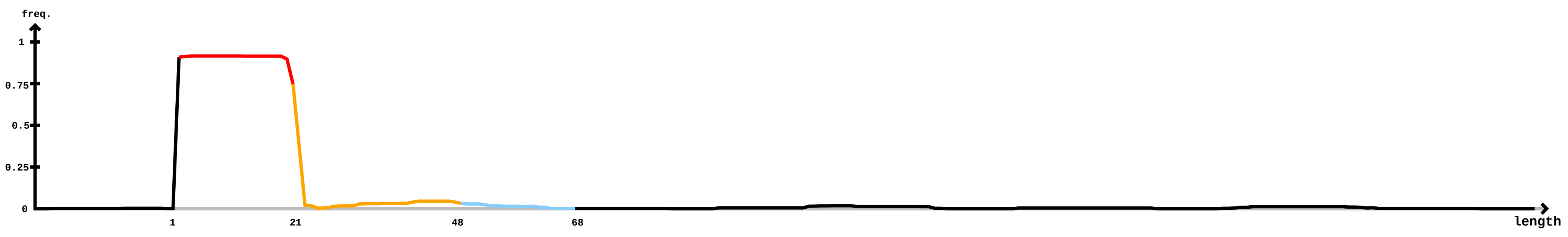

Mature Star

```
5'- uacuuguggacacaaaaacccauuagauucacgcacaaacuccuuuacuaggcagacuguagaaaaggagauuggaucugcauccaagcguuuuugacagaaaguaacaaucuucuucaccuucgcacaaagaugaagaagaugacggugccaugcuauggccugucaaacaaagaacauaucauucuccaggucagcaugcaauuugaagaaccucucauccauugaagcugcugucgacagagaugac -3'   exp
    (((((...(...(((((((.(.((.(((.((....(((.(((((((.(((.(.....).))).))))))).)))....)).))).))).))))))).)...)))))....(((((((((.(((.....))).)))))))))((((((.(((((...)))))))))))............((((.(((...(.(((((..((((.(((........)))...))))....))))).)....))))))).   reads   mm   sample
    .Gcuuguggacacaaaaacc.....................   2   1   LT-3
    .............aaaaacccauuagauucacU........   1   1   JCK-2
    .....................uuagauucacgcacaaac..   1   0   JCK-1
    .....................uuagauucacgcacaaac..   9   0   LCK-3
    .....................uuagauucacgcacaaac..   5   0   LT-3
    .....................uuagauucacgcacaaac..   2   0   JCK-2
    .....................uuagauucacgcacaaac..   5   0   LT-1
    .....................uuagauucacgcacaaacu.   14   0   JCK-3
    .....................uuagauucacgcacaaacu.   5   0   JCK-2
    .....................uuagauucacgcacaaacu.   21   0   LCK-1
    .....................uuagauucacgcacaaacu.   31   0   LCK-3
    .....................uuagauucacgcacaaacu.   24   0   LT-3
    .....................uuagauucacgcacaaaUu.   5   1   LCK-3
    .....................uuagauucacgcacaaacu.   23   0   LCK-2
    .....................uuagauucacgcacaaacu.   22   0   LT-2
    .....................uuagauucacgcacaaacu.   33   0   LT-1
    .....................uuagauucacgcacaaacu.   4   0   JCK-1
    .....................uuNgauucacgcacaaacu.   1   1   LT-2
    .....................uuagauucacgcacaaacuc   162   0   LT-2
    .....................uuagauucacgcacaaacuc   27   0   JCK-1
    .....................uuagauucacgcacaaacuc   1   0   JT-2
    .....................uuagauucacgcacaaacuc   19   0   JCK-3
    .....................uuagauucacgcacaaacuc   31   0   JCK-2
    .....................uuagauucacgcacaaacuc   142   0   LCK-3
    .....................uuagCuucacgcacaaacuc   1   1   LT-1
    .....................uuagauucacgcacaaacuc   104   0   LT-1
    .....................uuagauucacgcacaaacuU   2   1   LT-2
    .....................uuagauucacgcacaaacuU   7   1   LCK-3
    .....................uuagauucacgcacaaacuc   37   0   LCK-2
    .....................uuagauucacgcacaaacuU   5   1   LT-3
    .....................uuCgauucacgcacaaacuc   1   1   LCK-3
    .....................uuagauucCcgcacaaacuc   1   1   LT-1
    .....................uuagauucacgcacaaacuU   2   1   LT-1
    .....................uuagauucacgcCcaaacuc   1   1   LT-3
```

```
                      Mature                                         Star
uacuuguggacacaaaaacccauuagauucacgcacaaacuccuuuacuaggcagacuguagaaaaggagauuggaucugcauccaagcguuuuugacagaaaguaacaaucuucuucaccuucgcacaaagaugaagaagaugacggugccaugcuauggccugucaaacaaagaacauaucauucuccaggucagcaugcaauuugaagaaccucucauccauugaagcugcugucgacagagaugac
......................uuagauucacgcacaaacuc................................................................................................................................................................................................................    33    0    LCK-1
......................uuagauucacgcacaaacuc................................................................................................................................................................................................................    4    0    JT-3
......................uuCgauucacgcacaaacuc................................................................................................................................................................................................................    1    1    LT-1
......................uuagCuucacgcacaaacuc................................................................................................................................................................................................................    1    1    LT-3
......................uuagauucacgcacaaacuc................................................................................................................................................................................................................    57    0    JT-1
......................uuagauucacgcacaaacuc................................................................................................................................................................................................................    156    0    LT-3
......................uuagCuucacgcacaaacuc................................................................................................................................................................................................................    1    1    LT-2
......................uuagauucacgcacaaacucU...............................................................................................................................................................................................................    8    1    JCK-1
......................uuagauucacgcacaaacuUc...............................................................................................................................................................................................................    3    1    LT-1
......................uuagauucacgcacaaacucU...............................................................................................................................................................................................................    11    1    LT-1
......................uuagauucacgcacaaacuUc...............................................................................................................................................................................................................    2    1    LT-2
......................uuagauucacgcacaaacucc...............................................................................................................................................................................................................    1    0    LT-3
......................uuagauucacgcacaaacucA...............................................................................................................................................................................................................    1    1    JCK-3
......................uuagauucacgcacaaacucU...............................................................................................................................................................................................................    14    1    LT-3
......................uuagauucacgcacaaacucU...............................................................................................................................................................................................................    8    1    JCK-3
......................uuagauucacgcacaaacucU...............................................................................................................................................................................................................    7    1    LCK-3
......................uuagauucacgcacaaacuUc...............................................................................................................................................................................................................    1    1    JCK-3
......................uuagauucacgcacaaacucN...............................................................................................................................................................................................................    1    1    JCK-1
......................uuagauucacgcacaaacuUc...............................................................................................................................................................................................................    1    1    JCK-2
......................uuagauucacgcacaaacucA...............................................................................................................................................................................................................    1    1    LCK-3
......................uuagauucacgcacaaacucc...............................................................................................................................................................................................................    1    0    JCK-1
......................uuagauucacgcacaaacucU...............................................................................................................................................................................................................    26    1    LT-2
......................uuagauucacgcacaaacucA...............................................................................................................................................................................................................    3    1    LT-1
......................uuagauucacgcacaaacucU...............................................................................................................................................................................................................    6    1    JCK-2
......................uuagauucacgcacaaacucUu..............................................................................................................................................................................................................    1    1    LCK-3
......................uuagauucacgcacaaacucUu..............................................................................................................................................................................................................    1    1    LT-3
......................uuagauucacgcacaaacucGuu.............................................................................................................................................................................................................    2    1    LT-1
......................uuagauucacgcacaaacucGuu.............................................................................................................................................................................................................    9    1    JT-1
......................uuagauucacgcacaaacucUuu.............................................................................................................................................................................................................    6    1    LT-3
......................uuagauucacgcacaaacucUuu.............................................................................................................................................................................................................    1    1    LT-1
......................uuagauucacgcacaaacucUuuu............................................................................................................................................................................................................    1    1    JCK-3
......................uuagauucacgcacaaacucUuuu............................................................................................................................................................................................................    4    1    JCK-1
.......................uagauucacgcacaaacu.................................................................................................................................................................................................................    1    0    LT-2
.......................uagauucacgcacaaacuc................................................................................................................................................................................................................    2    0    LT-3
.......................uagauucacgcacaaacuc................................................................................................................................................................................................................    1    0    LCK-3
........................agauucacgcacaaacuc................................................................................................................................................................................................................    2    0    JCK-3
........................agauucacgcacaaacuc................................................................................................................................................................................................................    2    0    JCK-1
..............................................acuaggcagacuguagaaaagg......................................................................................................................................................................................    4    0    LT-1
..............................................acuaggcagacuguagaaaagga.....................................................................................................................................................................................    1    0    LT-3
...............................................cuaggcagacuguagaaaagg......................................................................................................................................................................................    1    0    JCK-1
...............................................cuaggcagacuguagaaaagg......................................................................................................................................................................................    2    0    LT-2
...............................................cuaggcagacuguagaaaaggag....................................................................................................................................................................................    1    0    JCK-2
................................................uaggcagacuguagaaaagga.....................................................................................................................................................................................    6    0    JT-3
................................................uaggcagacuguagaaaagga.....................................................................................................................................................................................    3    0    JT-2
.................................................aggcagacuguagaaaaggag....................................................................................................................................................................................    1    0    JCK-2
.................................................aggcagacuguagaaaaggag....................................................................................................................................................................................    1    0    LT-3
.................................................aggcagacuguagaaaaggag....................................................................................................................................................................................    1    0    JCK-1
....................................................cagacuguagaaaaggagauu.................................................................................................................................................................................    6    0    LCK-3
....................................................cagacuguagaaaaggagauug................................................................................................................................................................................    3    0    JCK-2
....................................................cagacuguagaaaaggagauug................................................................................................................................................................................    2    0    JCK-1
....................................................cagacuguagaaaaggagauugga..............................................................................................................................................................................    2    0    JCK-1
.....................................................agacuguagaaaaggagauug................................................................................................................................................................................    1    0    JCK-2
.....................................................agacuguagaaaaggagauug................................................................................................................................................................................    2    0    JCK-1
........................................................cuguagaaaaggagauuggaC.............................................................................................................................................................................    1    1    JCK-2
...........................................................uagaaaaggagauuggaucG...........................................................................................................................................................................    2    1    LT-2
.............................................................gaaaaggagauuggaucugca........................................................................................................................................................................    1    0    LT-1
.............................................................gaaaaggagauuggaucugca........................................................................................................................................................................    6    0    LCK-3
.............................................................gaaaaggagauuggaucugcauc......................................................................................................................................................................    1    0    JCK-2
.............................................................gaaaaggagauuggaucugcauc......................................................................................................................................................................    1    0    JCK-3
..............................................................aaaaggagauuggaucugcauc......................................................................................................................................................................    2    0    LT-1
..............................................................aaaaggagauuggaucugcauc......................................................................................................................................................................    5    0    LCK-3
.................................................................................auccaagcguuuuugacagaaag..................................................................................................................................................    2    0    JCK-1
................................................................................................................uucuucaccuucgcacaaagaug...................................................................................................................    6    0    LCK-3
...............................................................................................................................caaagaugaagaagaugacgg......................................................................................................    5    0    JCK-3
...............................................................................................................................caaagaugaagaagaugacgg......................................................................................................    3    0    LCK-3
...............................................................................................................................caaagaugaagaagaugacgg......................................................................................................    1    0    LT-3
...............................................................................................................................caaagaugaagaagaugacgg......................................................................................................    3    0    JCK-2
................................................................................................................................aaagaugaagaagaugac........................................................................................................    1    0    JCK-3
.................................................................................................................................aagaugaagaagaugacggug....................................................................................................    2    0    JCK-3
...................................................................................................................................gaugaagaagaugacUgug....................................................................................................    1    1    LT-1
```

Mature **Star**

```
uacuuguggacacaaaaacccauuagauucacgcacaaacuccuuuacuaggcagacuguagaaaaggagauuggaucugcauccaagcguuuuugacagaaaguaacaaucuucuucaccuucgcacaaagaugaagaagaugacggugccaugcuauggccugucaaacaaagaacauaucauucuccaggucagcaugcaauuugaagaaccucucauccauugaagcugcugucgacagagaugac
..................................................................................................................................................................cugucaaacaaagaacauaucau.................................................................	5	0	JCK-2
....................................................................................................................................................................................................gcaugcaauuugaagaaccuc.................................	1	0	JCK-2
....................................................................................................................................................................................................gcaugcaauuugaagaaccuc.................................	1	0	LT-1
....................................................................................................................................................................................................gcaugcaauuugaagaaccuc.................................	1	0	JCK-1
......................................................................................................................................................................................................augcaauuugaagaaccucuc...............................	1	0	JCK-3
......................................................................................................................................................................................................augcaauuugaagaaccucuc...............................	1	0	JCK-1
.......................................................................................................................................................................................................ugcaauuugaagaaccucuca..............................	1	0	JCK-3
.......................................................................................................................................................................................................ugcaauuugaagaaccucuca..............................	2	0	LT-1
.......................................................................................................................................................................................................ugcaauuugaagaaccucuca..............................	1	0	JCK-2
.......................................................................................................................................................................................................ugcaauuugaagaaccucuca..............................	1	0	JCK-1
.........................................................................................................................................................................................................caauuugaagaaccucucauc............................	2	0	LT-3
.........................................................................................................................................................................................................caauuugaagaaccucucauc............................	1	0	JCK-1
.........................................................................................................................................................................................................caauuugaagaaccucucauc............................	1	0	JCK-3
.........................................................................................................................................................................................................caauuugaagaaccucucauc............................	1	0	JCK-2
.............................................................................................................................................................................................................................ccauugaagcugcugucg...........	1	0	JCK-2
.............................................................................................................................................................................................................................ccauugaagcugcugucg...........	1	0	JCK-1
```
